# Editorial: Heterologous Immunity: Implications and Applications in Vaccines and Immunotherapies

**DOI:** 10.3389/fimmu.2020.01408

**Published:** 2020-07-07

**Authors:** Shakti Singh, Stephanie K. Yanow, Babita Agrawal

**Affiliations:** ^1^The Lundquist Institute for Biomedical Innovation, Harbor-UCLA Medical Center, Torrance, CA, United States; ^2^School of Public Health, University of Alberta, Edmonton, AB, Canada; ^3^Department of Surgery, Faculty of Medicine and Dentistry, University of Alberta, Edmonton, AB, Canada

**Keywords:** heterologous immunity, trained immunity, vaccines, immunotherapies, autoimmunity

Heterologous immunity is defined as immunity that can be induced by a pathogen or antigen against another unrelated pathogen, antigen or even an autologous antigen. The applications of “heterologous immunity” can be traced back to the first vaccine by Edward Jenner in the late seventeenth century where he used a cowpox virus to immunize against smallpox infection. Following this, the concept of heterologous immunity and its implications for vaccine development and immunopathogenesis were largely neglected until the late 20th century. However, epidemiological studies involving various vaccinated populations continued to suggest the existence of heterologous immunity. With the availability of modern bioinformatics and immunological tools, heterologous immunity among several pathogens has now been recognized and the immunomodulatory role in protective immunity and immunopathogenesis is being studied extensively. The topic “Heterologous Immunity: Implications and Applications in Vaccines and Immunotherapies” covers current progress in the field of heterologous immunity and is a collection of 13 articles that includes reviews, minireviews and original research.

The review article by Agrawal summarized the mechanism of heterologous immunity and its implications in pathogenesis and infectious disease outcomes. The mechanism of cross-reactive antibody or T cell immunity among related or un-related pathogens lies in the promiscuous nature of the interaction of B cell receptor and T cell receptor for epitope recognition. As a result, exposure to microbiota and various pathogens, and routine vaccination programs induce a considerable pool of cross-reactive T cells as well as antibodies able to respond to a completely different pathogen. The author further describes heterologous immunity among a broad range of pathogens and discusses its plausible role in natural resistance and modulation of the course of infection.

Balz et al. outline the consequences of heterologous immunity in antiviral immunity. In this review, the authors highlight important applications of heterologous immunity in vaccine development. Accordingly, for influenza viruses, they suggest that future vaccine development efforts should be toward a universal vaccine focused on broader T cell responses. The authors further discuss the public health implications of heterologous immunity and suggest that more knowledge on heterologous immunity among various pathogens can provide insight into the repurposing of existing vaccines. They also point out factors that influence the clinical outcomes of heterologous immunity and specifically mention the benefits of measles and vaccinia vaccination in this regard.

Flaviviruses are among the most common viral infections in tropical and subtropical countries. Heterologous immune responses among Flaviviruses are well-documented. Rathore and St. John review cross-reactive antibody and T cell immunity directed toward Flaviviruses and their role in protection and the development of immunopathologies. The authors also detail rational vaccine design from the viewpoint of cross-reactive immunity.

Malaria, a mosquito-borne disease, is caused by the *Plasmodium* parasite and is among the leading causes of death in endemic areas. In a comprehensive review, Mitran and Yanow describe cross-reactive antibody and T cell immunity among distinct species of *Plasmodium* that have been reported both in human populations and in experimental animal models. The authors define potential antigen targets for cross-species vaccine design in the context of heterologous immunity that would encompass evolutionarily conserved epitopes.

Heterologous immunity is particularly important from an immunopathology standpoint where it can negatively modulate the immune responses against another pathogen. This can occur through the induction of biased non-protective cross-reactive antibodies and T cells. The research article by Tang et al. highlights this phenomenon in a study of infections in mice with mixed-species of malaria parasites. The authors show that co-infection of mice with *Plasmodium yoelii* with either *Plasmodium vinckei* or *Plasmodium chabaudi* increased virulence (100% mortality) compared to mono-infections where mortality was significantly lower (40% with *P. chabaudi* and no mortality with *P. yoelii* or *P. vinckei)*. The authors discuss the role of immune competition/modulation on parasite virulence and the fitness costs of co-infection on transmission to mosquitoes.

Apart from vaccines, heterologous immunity is an attractive approach for the development of immunotherapeutic monoclonal antibodies. Youssef et al. identified a monoclonal IgM antibody against fungus *Candida albicans*' Hyr1 antigen that cross-reacts with the Gram-negative bacteria *Acinetobacter baumannii* and *Klebsiella pneumoniae*. Moreover, this antibody protects mice from lethal bacteremia. This study shows that a single monoclonal antibody can provide cross-kingdom protection against multiple microorganisms.

The T cell receptor has remarkable promiscuity toward its antigen epitopes and this feature is responsible for the generation of cross-reactive T cells. Cross-reactive T cells have several clinical implications for immunopathogenesis, autoimmunity, and alloreactivity and graft rejection. For example, Rowntree et al. published an elegant study where CD8^+^ T cells specific for different viral epitopes (EBV, CMV, and HIV-1) cross-reacted with HLA-B27 allotypes in a hierarchical manner. This study clearly shows that heterologous immunity has clinical implications wherein antiviral T cells induced against viral pathogens could contribute to adverse outcomes in allogeneic transplantation. It also implies that exposure of a host to multiple viral infections could lead to a more complex allo-reactivity.

The advent of newer T cell-based therapies (such as CAR-T cells) and their widespread clinical uses can be vulnerable to the cross-reactive nature of the T cell receptors. A study by Soon et al. shows that a therapeutic T cell specific to a hepatitis E virus epitope cross-recognized an epitope from an apoptosis-related autoantigen in the host, which could potentially lead to autoimmunity. Most importantly, the authors characterized a molecular signature of a multiple-glycine motif in the CDR3 region of the TCR β chain which may allow greater structural flexibility with a minimum energy threshold required for promiscuity. Therefore, such T cell-based therapies should be evaluated for any off-target cross-reactivity.

Heterologous immunity is also relevant for vaccine development against highly diverse pathogens where cross-strain reactivity or cross-species protection is critically desirable. As an example, current vaccine development efforts against influenza viruses are focused on developing a broadly cross-reactive universal vaccine. These efforts employ modern *in silico* and immunological tools to design cross-reactive, conserved antigen epitopes as well as the use of a suitable adjuvant formulation that favors the induction of antibodies and T cells with broader specificities. Nguyen et al. show that the Pandemic H1N1 vaccine formulated with poly-γ-glutamic acid (PGA)/Alum complex provided cross-protection against heterologous influenza viral strains: A/Puerto Rico/8/34 (H1N1) and A/Hong Kong/1/1968 (H3N2)]. In a similar study, Luo et al. showed that the H7N9 vaccine formulated with STING agonist cGAMP could provide effective cross-protection against H1N1, H3N2, and H9N2 influenza viruses in mice. Finally, Lee et al. showed that mincle and STING-stimulating adjuvant formulated with a foot and mouth disease virus vaccine induced a robust and long-lasting cellular and humoral memory response across diverse species in mice, cattle and pigs. These studies suggest that the use of new, advanced adjuvant formulations will not only induce antibodies and T cells with broader specificities for greatly improved immunity but also overcome disparities in immunogenicity of a vaccine across species.

Heterologous immunity is not only limited to adaptive immunity. Innate immunity triggered by one pathogen or vaccine can protect against an unrelated pathogen. This is often called “trained immunity” and is represented by innate immune cells. Trained immunity arises due to epigenetic reprogramming of innate immune cells such as macrophages upon exposure to infection/vaccine. Covián et al. reviewed BCG vaccine-induced trained innate immunity and its role in cross-protection and heterologous effects.

Furthermore, macrophages play a distinct role in heterologous immunity because they have different functional stages at various points in the course of an infection. This process results in an intrinsic functional imprinting/training of macrophages by the invading pathogen. Connolly and Hussell review several aspects of this training of macrophages and the influence of Type 1 interferons on the alveolar macrophage. The authors also discuss how influenza virus-mediated production of Type 1 interferon alters the functional response of macrophages toward bacterial superinfection.

Collectively, these reviews and original research articles provide a comprehensive overview of the various dimensions of heterologous immunity. They support the outlook that heterologous immunity should be an important aspect in the design, testing and development of vaccines and immunotherapeutics. In addition, the influence of heterologous immunity should be carefully examined on clinical outcomes of infections and autoimmunity.

## Author Contributions

SS wrote the manuscript. BA and SY performed critical revision and editing. All authors share equal intellectual contribution to this work and approved it for publication.

## Conflict of Interest

The authors declare that the research was conducted in the absence of any commercial or financial relationships that could be construed as a potential conflict of interest.

